# Experiences of informal caregiving among older lesbian and gay adults in Australia

**DOI:** 10.1111/ajag.13076

**Published:** 2022-05-12

**Authors:** Andrea Waling, Anthony Lyons, Beatrice Alba, Victor Minichiello, Catherine Barrett, Mark Hughes, Karen Fredriksen‐Goldsen, Samantha Edmonds, Nicky Bath

**Affiliations:** ^1^ School of Psychology and Public Health, Australian Research Centre in Sex, Health and Society La Trobe University Melbourne Victoria Australia; ^2^ School of Psychology Deakin University Melbourne Victoria Australia; ^3^ School of Justice Queensland University of Technology Brisbane Queensland Australia; ^4^ Alice's Garage Melbourne Victoria Australia; ^5^ Faculty of Health Southern Cross University Gold Coast Queensland Australia; ^6^ School of Social Work University of Washington Seattle Washington USA; ^7^ Ageing with Pride Sydney New South Wales Australia; ^8^ LGBTIQ+ Health Australia Sydney New South Wales Australia

**Keywords:** aging, caregivers, gays, lesbians, social support

## Abstract

**Objective:**

This study examined older lesbian and gay adults' experiences regarding informal caregiving, including challenges and positive aspects of caregiving.

**Methods:**

Interviews were conducted with 16 lesbian women and gay men in Australia, aged 60+, who were engaged in informal caregiving. Analyses involved a qualitative thematic approach.

**Results:**

Most participants were caring for a friend or partner and regarded caregiving as a form of love and did not seek external support despite noting several impacts. Some participants found that they too were beginning to require care. For some, formal care was being considered, but with a degree of reluctance.

**Conclusions:**

Older lesbian and gay adult caregivers experience a range of challenges and support needs in relation to their experiences with the caregiving role. This research highlights a need for ensuring that caregiving policies and practices be responsive to the experiences and challenges faced by older lesbian and gay people.

**Practice Impact**

This research highlights a need for ensuring that policies and practices aimed at supporting caregivers also consider the experiences and challenges faced by older lesbian and gay people. This may include access to culturally safe and inclusive support services, as well as educating or encouraging caregivers about seeking financial and other types of support where applicable.

## 1 INTRODUCTION

Informal caregiving plays a crucial role in supporting many older people in Australia and across the globe.^1^, ^2^, ^3^ Often, it is older people themselves who provide care to others, such as a relationship partner, friend or family member.^1^, ^2^, ^3^ In some cases, caregivers take on responsibilities that impact other areas of their lives, such as their health and well‐being.^3^, ^4^

While anyone providing care may face challenges,^3^, ^4^ older lesbian and gay adults often face a range of distinct difficulties.^5^, ^6^ US, UK and Canadian studies have noted several themes emerging from qualitative explorations of lesbian and gay caregiving, including engagement with health services that resulted in experiences of discrimination,^7^ concerns in ‘outing’ oneself or the person receiving care when accessing services,^8^ friendships often being the only source of care^9^ and decline in well‐being for lesbian and gay caregivers.^10^ It is important to note that studies have also found that older lesbian and gay adults report many positive aspects of providing care, such as a sense of fulfilment, and showing love and commitment to those for whom they provide that care.^11^

Despite studies conducted abroad, research on informal caregiving among older lesbian and gay adults in Australia has been limited. Some studies have focused on the roles of caregivers regarding specific health conditions, such as dementia,^12^ and on discrimination when accessing health services.^13^, ^14^ A recent quantitative study noted that older lesbian and gay adults were more likely to experience poorer physical and mental health if they were caring for someone who identified as lesbian, gay, bisexual, trans or intersex (LGBTI) compared to caring for a non‐LGBTI person,^15^ possibly due to additional challenges related to discrimination.

While not directly related to caregiving, a few Australian studies have also noted lesbian and gay people's concerns about accessing health and aged care services.^16^, ^17^, ^18^, ^19^, ^20^, ^21^ Such concerns have included whether services were culturally safe, inclusive and had undergone appropriate LGBTI‐inclusive practice training for LGBTI people.^16^, ^17^, ^18^, ^19^, ^20^, ^21^ Some studies have also considered networks of care within lesbian and gay communities.^8^, ^22^ These studies explored varying aspects in which an older lesbian or gay person may receive and experience care, as well as the ways in which they may become engaged as caregivers.^22^ It is also worth noting for context that, in Australia, caregivers who meet eligibility requirements can seek a Carer Allowance, a stipend of up to AUD $136.50 per fortnight (at time of publication) that provides supplemental income to support caregiving. In‐home care services (e.g. domestic assistance and medical treatments) are also available to support individuals who require care in their home.

It is important to understand different ways this population perceives, understands and engages with caring roles, including some of the key challenges. This is useful for service providers to gain an understanding of strengths and challenges faced by older lesbian and gay adults. This study reports on 16 semi‐structured interviews with lesbian and gay adults aged 60 years and older in Australia. Our main aim was to examine the ways participants make sense of, and engage in, informal care for their partners, friends and family.

## 2 METHODS

Qualitative, semi‐structured interviews were chosen to allow for deeper insights into the diverse lived experiences of older lesbian and gay adults.^23^ Participants were recruited from a national survey (online and paper) in 2017 that examined diverse aspects of the health and well‐being of gender and sexually diverse populations.^13^, ^14^, ^15^ At the end of the survey, they were able to provide their contact details for an interview. In total, 368 lesbian women and gay men aged 60 years and older volunteered for an interview. Participants also provided their gender identity and sexual orientation and were assigned to groups based on this information. An online number generator was used to randomly select participants, who were subsequently contacted by email with a participant information and consent form prior to scheduling an interview. Interviews were conducted from September 2017 to December 2017, with ethics approval provided by La Trobe University Human Research Ethics Committee (S17/088).

In total, 33 interviews were conducted by the first‐named author. Interviews focused on diverse topics related to the health and well‐being of older lesbian and gay adults, including coming out, health concerns, accessing health and social care services,^13^ support networks and caregiving. Of these interviews, 16 involved participants who indicated they were engaged in some form of caregiving when asked if they were supporting anyone in an informal capacity. We referred to caregiving in a relatively broad sense, where people were providing care to someone who requires some form of support, and examples were given such as health needs or mobility. This enabled us to capture a range of potentially different caregiving practices involving different types of relationships between caregivers and care receivers. In this study, we focus on the above‐mentioned 16 participants, comprising 9 lesbian women and 7 gay men, all of whom were cisgender. Interviews were conducted via telephone. These were 45 to 60 min in length, audio‐recorded and externally transcribed by a professional agency. All transcripts were verified and anonymised, with pseudonyms assigned to participants.

Thematic analysis was conducted following Braun and Clarke's procedures.^24^ Transcriptions were loaded into NVivo, a qualitative analysis software programme. Stage 1 was conducted by the first‐named author who familiarised themselves with the data. A priori categories were used to determine the descriptive codes, and transcripts were coded by AW. Coding of random transcripts was reviewed by BA to assess trustworthiness. For this study, AW took the descriptive code ‘caregiving experiences’ to explore and analyse the emerging latent themes (stage 3). These themes were reviewed with the rest of the research team to ensure relevance to the main aim of the study (stage 4). Themes were then defined to better understand caregiving experiences of older lesbian and gay adults in Australia (stage 5). Findings were then written and reviewed (stage 6).

## 3 RESULTS

Table 1 summarises the demographics of the 16 participants. Most were in a partner relationship, and the sample had a median age of 66 years. A majority were retired, resided in urban areas of Australia and were not engaging with an in‐home aged care service. Over half indicated they did not have children. Most were caring for either a friend (8) (i.e. someone they would actively socialise with who was not considered a family member) or partner (5), but some were caring for a biological family member (2) (e.g. parent and sibling), neighbour (1) or a child with high care needs (1). Types of care included general tasks, house cleaning and cooking, social check‐ins and responding to high care needs such as physical and mental support.

**TABLE 1:** Demographic characteristics (*N* = 16)

Demographic characteristic	*N*	%
Age		
60–65	8	50%
66–70	6	38%
71–75	2	13%
Gender		
Men	7	44%
Women	9	56%
Sexuality		
Gay	7	44%
Lesbian	9	56%
Current relationship status		
Single	3	19%
Partnered	13	81%
Have children		
Yes	6	38%
No	10	62%
Who they were caring for		
Child with high care needs	1	6%
Family	2	13%
Friend	7	44%
Neighbour	1	6%
Partner	5	31%
Types of care^a^		
Household tasks (e.g. cleaning, cooking, gardening and/or home maintenance, finances, shopping/running errands)	9	56%
Mobility assistance (e.g. transportation, support with exercise or movement)	8	50%
Personal high care needs (e.g. bathing, feeding, cleaning and administering medications)	6	38%
Social and emotional support (e.g. socialising and check‐ins)	12	75%
Advocacy and support with services (e.g. filling out forms and contact with services)	4	25%
State or territory of residence		
Australian Capital Territory	1	6%
New South Wales	4	25%
Queensland	3	19%
South Australia	2	13%
Victoria	5	31%
Western Australia	1	6%
Residential area		
Urban	11	69%
Regional	3	19%
Rural	2	13%
Employment status		
Retired	9	56%
Working	7	44%
Receiving home‐care		
Yes	2	13%
No	14	88%
Education		
High school	3	19%
University	12	75%
Not known	1	6%

^a^ Participants often engaged in more than one type of care. For this reason, percentages do not add up to 100.

Participants noted estrangement from family and children from previous marriages due to experiences of homophobic discrimination and/or rejection. As a result, many engaged with friends from the LGBTIQ+ community and relationship partners for support and caring needs. Four overarching themes were identified: (1) caring as a labour of love; (2) physical, emotional and social impacts of caregiving; (3) needing care themselves; and (4) engaging with formal care services. As mentioned, participants were caring for a variety of people. There were no apparent differences in the women and men's caregiving patterns, so we report on both groups together. Where relevant, we attend to differences in experiences between caring for a partner, friend, neighbour or family member. For each quote, we provide the participant's pseudonym, gender and sexual orientation, and their relationship to the person for whom they were caring.

### 3.1 Caring as a labour of love

Participants often framed their caregiving duties as a form of love, particularly for romantic partners. Caring as love was evident in participants' capacity to be mindful of the emotional and other needs of relationship partners to whom they were providing care and being the best suited for this. For example, Jean (61, lesbian woman, partner) was caring for her partner whose condition causes extreme fatigue: *‘“I'll stay quiet while you [her partner] have a sleep,” that is my big support thing’*. Percy (63, gay man, partner) reported that he was very careful about maintaining his partner's dignity as his partner dealt with incontinence issues: *‘I have to deal with that discreetly. I don't want to embarrass him’*. Similarly, Cecilia (69, lesbian woman, partner) noted that while her experience of caring for her partner was *‘full on’*, it was *‘where I wanted to be’*.

This perspective was not limited to partners and was also noted by those caring for either a family member or friend. As a result, many did not feel the need to be financially supported for this work (e.g. via the Carer Allowance), particularly those supporting friends, neighbours and family. Patrick (69, gay man, sibling), who was caring for his sister, mentioned that while he was eligible for the Carer Allowance, he was not interested in accessing it because he was *‘quite happy to just do it because she's my sister and I love her’*. Similarly, Cameron (66, gay man, friends) who cared for a variety of friends did not *‘find it a problem extending myself to anyone’*. Indeed, very few participants who were engaging in some form of caregiving, whether for partners, friends or family, took up the opportunity for financial support.

### 3.2 Physical, emotional and social impacts of caring

As care was often framed as a labour of love, participants were reluctant to draw attention to difficulties encountered in caregiving. Nevertheless, some challenges were noted, which were particularly salient for those caring for partners. Physical difficulties were often referred to, especially in relation to caregiving participants' own bodies beginning to weaken with either age‐ or health‐related issues, such as decreased energy, mobility and capacity. Patrick (69, gay man, sibling) provided house cleaning for his sister who has a long‐term health condition, but expressed that he stopped as *‘I just found it harder and harder to do, and it's the same with my own place, I find it hard to keep things the way I used to’*. Emotional impacts included difficulties watching a partner suffer through pain and illness. Caregivers noted frustrations and reported that caregiving was impacting their own emotional well‐being. For example, Devon (69, gay man, partner) referred to becoming increasingly worried about his capacity to care for his ailing partner and noted feelings of impatience as a result: *‘I do tend to get a little bit impatient with him because I am worried and he does get a bit upset with me’*.

Others expressed feeling frustrated when partners were not helping themselves, such as Phoebe (66, lesbian woman, partner) who noted she was *‘badgering her [partner] when she had a turn’* when she felt her partner ignored warning signs of an impending heart attack after having had one previously. Some also reported being worried about leaving partners when they went out, such as Percy (63, gay man, partner) who noted, *‘there are lots of things I can do by myself…but it means I'm leaving [PARTNER] at home by himself, and that's a worry*’. Others indicated that they had limited time to themselves to engage in personal activities, with much of their time devoted to caregiving.

Some of these impacts were also expressed by those caring for either friends or family members. For example, Whitney (69, lesbian woman, friend), who was caring for an older friend, echoed these sentiments, noting that her own ageing meant she was *‘…getting physically tired… By the time I get home I'm physically exhausted’*. Whitney also reported emotional impacts, stating that *‘I'm probably not as patient as I should be’* when supporting her friend, and was considering ending her support. Indeed, those caring for family members or friends seemed more likely to consider ending support when it was impacting them, whereas those caring for partners demonstrated greater reluctance to stop providing care.

### 3.3 Needing care themselves

Participants reported concerns that, as they aged, they too may need care in the future. Some were unsure how they might receive care while still providing care to others. For example, Percy (63, gay man, partner) was caring for his partner with mental health challenges and had done so for the last few years. Recently, however, Percy was beginning to require his own medication treatment and support: *‘What I am worried about is I've been the care[giver] up to now, but fairly soon I'm going to need care myself, and I'm not sure whether [PARTNER]'s going to be up to [it]’*. Jean (61, lesbian woman, partner), who was the primary caregiver for her partner, shared a similar story. She recounted how a back injury left her having to care for herself as her partner was unable to do this for her: *‘Nobody could actually lift me or support me as I tried to walk…Even the slightest movement in the wrong direction could cause pain so I had to do it all myself*’.

In general, participants caring for partners were more likely to report concerns about the impact on caregiving if they needed care themselves, whereas those caring for friends or family appeared less concerned about this. Some also believed that their future support network would actively take on this role. For example, Debbie (65, lesbian woman, friends) noted that there had been discussions about her care from her broader family, but nothing was concrete: *‘I imagine that they'll be looking after me. They argue about who's going to get the pleasure of it’*. Similarly, Cameron (66, gay man, friends) expressed confidence that his friends would help him as he has done for them, but did not mention his partner as a potential caregiver.

### 3.4 Engaging with formal care services

In other papers based on the larger data set,^13^, ^14^ we have reported on older lesbian women's and gay men's concerns about having to use a formal care service for themselves, such as entering residential care, due to potential for hostility, discrimination or even violence from those who do not support lesbian and gay people. Building on this, we discuss here how caregivers felt about engaging with similar services on behalf of their partner. Sam (63, gay man, friends) reported this as a future concern, based on experiences of his heterosexual friends in a de facto relationship who *‘were getting married specifically because [FEMALE FRIEND] has a brain tumour and they'd experienced recently where the hospital did not recognise [MALE FRIEND] as her partner’*. Sam felt that if hospitals would not recognise the de facto status of heterosexual couples, they would not consider same‐sex partner status.

This also meant that participants who had partners requiring care were doing most of this work themselves rather than engaging a service for assistance or support. For example, Devon (69, gay man, partner) noted the increasing difficulties of caring for his partner due to his own declining health: *‘It is getting hard because I am worried about how much I can do for him’*. Percy (63, gay man, partner) echoed these sentiments, indicating that his partner's *‘mental health needs will come to the fore much more quickly than my physical decrepitude’* thus resulting in potential separation via residential care services.

Similarly, Danielle (64, lesbian woman, child) struggled with decisions about her son, who has high care needs related to a disability. She was concerned about placing her son in residential aged care, as there were limited other options available for people with disabilities and high care needs. Indeed, Danielle highlighted that her son is *‘young, you know for a nursing home…you know look I don't feel good about it, and we're slowly having talks about it’*. Mabel (60, lesbian woman, neighbour) noted that she and her partner cared for older heterosexual neighbours as appropriate services did not reach rural areas: *‘We've been looking after a neighbour who's got dementia, and his wife was told that they weren't eligible for [at‐home aged care] services, that we live too far out’*.

## 4 DISCUSSION

In this study, participants described a range of experiences as caregivers, including caring as a labour of love; the physical, emotional and social impacts of caregiving; needing care themselves; and considerations related to engaging with formal care services.

Many of these themes are reflected in general population, such as concerns about accessing formal care services, caring as a form of love and the personal toll of care on caregivers.^1^, ^2^, ^3^, ^11^ However, concerns about discrimination also arose in our findings, relating to the specific backgrounds of the participants, which can result in greater complexities older lesbian and gay adults' caregiving. Similar issues are found among other marginalised communities such as Indigenous and People of Colour communities, and people with disabilities and neurodivergencies.^25^, ^26^ While not reported in this study, other studies have also noted that informal caregiving could be a way to avoid potential experiences of discrimination in formal care settings.^4^, ^5^, ^8^, ^11^, ^13^, ^14^, ^16^ The broader history of discrimination towards lesbian women and gay men has resulted in a community‐driven imperative in which individuals tend toward taking care of each other and less toward engaging with formal services.^27^

The social restrictions put in place during the COVID‐19 pandemic might have further added to these issues. Providing support for others or accessing support themselves may have been more challenging due to COVID‐19 restrictions, and social support is known to be an important protective factor for mental health among lesbian and gay people^27^ and more recently among lesbian and gay caregivers.^28^ Further understanding of these potential issues would be required in future research, including attention to identifying and addressing specific challenges that older lesbian and gay caregivers may have in accessing support. Of particular importance would be further investigation of some notable findings from this study, such as not seeking financial support and caring for neighbours.

Taken together, the study findings have potential implications for policy and practice. The recent *Royal Commission into Aged Care Quality and Safety* ^29^ in Australia highlighted that LGBTI caregivers face additional challenges in accessing formal care services^5^ and studies have highlighted that LGBTI people are concerned about using services in fear of discrimination and violence.^13^, ^14^, ^16^ Finding ways to support caregivers will be important. This could include encouraging eligible caregivers to access financial support, ensuring in‐home care services are safe for older lesbian and gay people, and educating caregivers about services available to them. Additionally, some research has found that support groups may be helpful for caregivers.^30^ This may be especially important for preventing social isolation, which could be a particular challenge for some lesbian and gay people who feel marginalised or socially excluded.^4^, ^8^, ^10^, ^12^, ^15^, ^16^, ^17^

There were a few limitations to this work. The sample had a median age of 66 with very few over 75. Caregivers in the oldest age groups may have other experiences, and this would need to be explored in future research. This study was also limited to cisgender lesbian women and gay men, and it is likely that other groups, such as bisexual and trans and gender diverse people, have a range of different experiences. It is important that the experiences of these groups are also given dedicated attention in future work. Participants were also recruited from a survey that was not population representative. Due to a lack of census data, it is not currently possible to definitively determine population estimates for older lesbian and gay adults. As such, further work is needed to examine caregiving experiences in additional samples of older lesbian women and gay men to corroborate our findings and generate further insights. Additionally, understanding experiences of coping with caregiving and social isolation would be useful. The interviews in this study covered a range of topics, and qualitative studies that specifically focus on caregiving would be useful for delving more deeply into participants' experiences. Such research could focus more extensively on the histories, experiences and needs of caregivers, as well as the experiences of those who receive care, including the experiences of other groups such as bisexual and trans and gender diverse caregivers and care receivers.

## 5 CONCLUSIONS

This study provided insight into the experiences and needs of older lesbian and gay caregivers in Australia. While the majority were providing care to a friend or relationship partner, some were caring for family members or neighbours. There were many positive experiences but also a range of challenges, which are likely to be contextualised by long histories of stigma and barriers to accessing services. These findings may be useful for service providers, including in‐home services, for informing the design and implementation of policies and practices to support older lesbian and gay caregivers based on their perspectives, experiences and needs.

## CONFLICTS OF INTEREST

No conflicts of interest declared.

## Data Availability

Data are not available to be shared due to participants not providing consent to have their data shared.
